# Demographic differences in perceived effectiveness for policies to prevent school shootings: results from a representative survey in New Jersey

**DOI:** 10.1186/s40621-024-00520-6

**Published:** 2024-08-06

**Authors:** Michael Anestis, Jayna Moceri-Brooks, Allison Bond, Daniel Semenza

**Affiliations:** 1 New Jersey Gun Violence Research Center, Rutgers School of Public Health, Rutgers University, 683 Hoes Lane West, Piscataway, NJ 08854 USA; 2 Department of Urban-Global Public Health, Rutgers University, Piscataway, NJ USA; 3 Department of Psychology, Rutgers University, Piscataway, NJ USA; 4 Department of Sociology, Anthropology, and Criminal Justice, Rutgers University –Camden, Camden, NJ USA

## Abstract

**Objective:**

To determine what firearm policies New Jersey residents believe will prevent school shootings and the extent to which this varies by sex, firearm ownership status, and political affiliation.

**Methods:**

A representative sample of New Jersey residents (N = 1,018) was collected via the Eagleton Center on Public Interest Polling (ECPIP). Data were weighted to reflect the state's population. Participants were asked to rate how helpful they perceived different firearm-related policies to be for preventing school shootings.

**Results:**

Findings indicate that participants perceived universal and expanded background checks, increased mental health funding, and requiring a license for firearm purchases as most effective for preventing school shootings. Arming school personnel, prayer in schools, decreasing the number of entrances at schools, and secure storage requirements were viewed as less effective. Firearm ownership, sex, and political affiliation significantly influenced perceptions of the effectiveness of these policies.

**Conclusion:**

The study examined the perceived effectiveness of policies to prevent school shootings. The study highlights disparities and commonalities in policy support among different groups, emphasizing the importance of collective efforts to address gun violence in schools.

## Introduction

School shootings are a significant public health concern within the United States (US). The number of school shootings has risen steadily over the past seven years, with a startling spike after the start of the COVID-19 pandemic and firearm purchasing surge in 2020. Shootings on school grounds nearly tripled between 2019 and 2022, reaching a record high of 346 shootings in 2023 (National Center for Education Statistics 2023). These numbers include shootings in and around school environments, such as parking lots, sports fields, and on school buses. From 2000 to January 2024, there were 50 active shooter events in K-12 schools (National Center for Education Statistics 2023). The term “active shooter” is used by law enforcement to refer to an incident where one or more individuals are actively attempting to kill people in a populated area, often leading to a mass casualty event.

In response to tragedies such as the mass shooting in Uvalde, Texas, many states have advocated for and implemented policies to prevent firearm violence. The varying policies, however, are often viewed through a partisan or cultural lense—with different communities viewing different policies as potentially useful. These policies include restrictions on firearms and firearm ownership, school infrastructure modifications, and school programming directed towards mental and spiritual health.

### Public opinions on policies to reduce school shootings

Research demonstrates that most people within the US support legislation that focuses on the firearm owner rather than the firearm itself. For example, Burton et al. (2021) found that, among a national sample of US adults, the majority supported banning the sale of firearms to those with “mental illnesses,” requiring background checks for firearm purchases, and requiring a mandatory five-day waiting period for all firearm purchases. Most participants in this study also supported programs and infrastructure changes within schools, such as increasing mental health counseling, anti-bullying programs, metal detectors, security cameras, and controlled access in schools to prevent and/or thwart school shootings (Burton et al. 2021). However, the majority of participants did not support restricting the number or type of firearms owned (Burton et al. 2021). National polling data reflects these findings, showing that the majority of people in the US only favor restrictive firearm laws (e.g., background checks and age limits) that focus on the individual (not the firearm) and support for permissive firearm laws (e.g., arming teachers) is sharply divided by political leaning (Schaeffer 2023). In line with this, several states have passed permissive firearm laws as a way to mitigate school shootings (Stanford 2024). For example, in 2018, Virginia implemented a policy to allow K-12 teachers to carry firearms (Mancini et al. 2020). This policy was supported by nearly half of the residents of the State (Mancini et al. 2020). National polling on support for arming teachers reveals nearly the same level of support (43%) (Pew Research Center 2021). Another policy that has garnered support from a smaller subset of lawmakers is the return of prayer in schools to reduce firearm violence (Marsh 2023; Benen 2022).

When school shootings occur in the US, mental illness is often portrayed as the primary cause, despite evidence on the contrary (Tikkanen et al. 2020). Compared to Canada, the United States has similar rates of depression (US: 23%; CA: 20%) and emotional distress (US: 26%, CA: 27%) (Tikkanen et al. 2020). However, from 2009 to 2018, Canada had two instances of school shootings, while the US surpassed all other nations with 288 school shootings (World Population Review 2024). A notable difference between the countries is the availability of firearms. It is estimated that there are 7.1 million firearms in Canada (Government of Canada 2022) and over 494 million firearms in the US (Mascia and Brownless 2024). Despite this evidence, increased funding for mental health treatment continues to be viewed as an effective prevention strategy for school shootings. This may be an especially appealing policy for firearm owners, as they may perceive it as reducing school shootings, while not infringing on their Second Amendment rights.

### Public opinions on policies to reduce firearm violence in general

Most existing research related to firearm injury prevention policies are not specific to school shootings, but address firearm injury prevention more broadly. Research has examined demographic differences that impact the public’s support of firearm legislation. For example, among a nationally representative sample in the US, both firearm and non-firearm owners supported universal background checks, accountability for licensed dealers, safety training for concealed carry permit, improved reporting of mental illness, and firearm prohibitions for persons with domestic violence restraining orders (Barry et al. 2018). In the same study, non-firearm owners supported policies related to banning specific types of weapons and high capacity magazines to a greater degree than did firearm owners (Barry et al. 2018). In addition to differences based on firearm ownership status, support varied based on racial identity. Specifically, Black individuals supported several policies (e.g., restrictions on assault weapons, increasing the minimum age for firearm purchasing) to a greater degree than white individuals, while Hispanic individuals supported secure storage requirements to a greater degree than white individuals (Crifasi et al. 2021a).

Within the realm of firearm violence prevention, general support for a policy does not necessarily mean an individual believes that policy will be effective in preventing a specific form of firearm violence. Although conversations about support for firearm and school security legislation may be prompted by mass school shooting events, it is unclear from prior work whether support for specific forms of legislation is a sign that individuals believe such policies would be effective specifically in preventing school shootings.

Although research on support for firearm policies has been conducted on a national level, less work in this area has been done at state and local levels. New Jersey boasts a low school shooting rate and the third lowest firearm death rate in the country (Giffords Law Center 2024); however it remains unknown whether New Jersey residents align in their opinions with nationally representative samples. Given the landscape of relatively restrictive firearm policies in New Jersey, it is important to understand to what extent New Jersey residents believe the firearm policies in their state are preventing school shootings and if their beliefs differ by demographic factors like sex, firearm ownership status, and political affiliation.

Much of the current research on firearm policy support has examined demographic variables associated with support for firearm policies. However, no study has examined what individuals think is *effective* for reducing school shootings, regardless of whether they personally support the policy. The present study seeks to fill this gap by determining demographic differences in the perceived effectiveness of commonly proposed policies for preventing school mass shootings among a representative sample of New Jersey residents. The study aims were to identify which firearm policies individuals believe will prevent school shootings and to analyze the extent to which the perceived effectiveness of policies in preventing school mass shootings varies by sex, firearm ownership status, and political leanings. Findings from this study will provide insight into factors that may influence individuals’ perceptions regarding the efficacy of firearm policies to prevent active shooter incidents at schools. Such understanding can help provide clarity for gaps between perception of effectiveness and empirical support for specific policies.

## Method

See Table 1 for sample descriptive statistics.

**Table 1:** Sample characteristics

	% (n)
Sex	
*Male*	47.6 (484)
*Female*	51.6 (526)
Age	
*18-34*	26.2 (267)
*35-49*	24.2 (247)
*50-64*	28.0 (285)
*65+*	21.3 (216)
Racial identity	
*White*	60.7 (618)
*Black*	12.3 (125)
*Hispanic*	8.9 (90)
*Other*	15.8 (161)
Education	
*High school or less*	28.9 (294)
*Some college*	30.8 (314)
*College graduate*	21.7 (221)
*Graduate work*	18.3 (186)
Annual household income	
*< $75,000*	37.7 (384)
*$75,000–< $150,000*	31.4 (320)
*$150,000+*	18.2 (186)
Firearms kept in or around home	
*Yes*	19.8 (202)
*No*	70.4 (717)
*Unsure*	4.9 (50)
*Refuse to answer*	4.3 (44)
Political affiliation	
*Democrat*	41.1 (348)
*Independent*	34.9 (296)
*Republican*	24.0 (203)

### Participants and procedures

All procedures were approved by the Institional Review Board (IRB) at Rutgers Biomedical and Health Sciences and the study was performed in accordance with the ethical standards as laid down in the 1964 Declaration of Helsinki and its later amemdments. Participants (N=1,018) were adult New Jersey residents, recruited by the Eagleton Center on Public Interest Polling (ECPIP) during an omnibus statewide survey conducted July 18 through July 27, 2022. ECPIP utilized probability-based sampling, and utilizing random digit dialing, conducted cell phone interviews (27%), landline interviews (24%), and text-to-web survey completion (50%). Inclusion criteria were being age 18 or above and residing in New Jersey.

Data were weighted to be representative of the residential adult population of New Jersey, balanced to match parameters for sex, age, education, race/ethnicity, region, and phone use. Weighting was conducted in two stages. The first step corrects for different probabilities of selection across the telephone samples associated with the number of adults in each household and each respondent’s telephone usage patterns, while also accounting for the overlap in landline and cell phone sample frames and the relative sizes of each frame and sample. The second stage balances sample demographics to match target population benchmarks, with weights trimmed to prevent individual interviews from having disproportionate influence on survey estimates. The adjusted margin of error in this sample—factoring in the design effect (1.57) —was +/- 3.8 percentage points at a 95% confidence interval.

The New Jersey Gun Violence Research Center (GVRC) purchased a total of 3 minutes of survey in the ECPIP omnibus state poll. The GVRC included a variety of items related to gun violence prevention and were able to access the final data related to these items, other gun violence-related items designed by ECPIP, and the sample demographics. The GVRC had no access to any other data collected from this sample.

### Measures

Firearm access in the home was assessed via a single item that asked “Are there any firearms typically kept in or around your home?” Individuals who indicated that they do not know or who refused to answer were not included in analyses leveraging this variable.

Political affiliation was assessed by asking participants “In politics today, do you consider yourself a Democrat, Republican, Independent, or something else?” For conceptual clarity, only individuals who endorsed Democrat, Republican, or Independent were included in analyses leveraging this variable.

Belief in the effectiveness of various policies to prevent school shootings were assessed via a series of items designed by the study team. Due to time constraints for the survey, half of the sample was randomized to receive one selection of policy questions and the other half received the other selection of policy questions^1^. In each case, participants were presented with the following question: “How helpful do you think the following policies are or would be for preventing mass school shootings like the recent tragedy in Uvalde, Texas?”

The first set of policy questions included the following options: “Requiring background checks for all firearm sales,” “Requiring background checks to ask more detailed background information about potential firearm purchasers,” “Reducing the number of entrances at schools,” “Providing teachers and other school personnel with firearms,” “Banning AR-15s and similar ‘assault’-style weapons,” and “banning the use of high-capacity magazines that enable an individual to fire a larger amount of ammunition before needing to reload.”

The second set of policy questions included the following items: “Increasing funding for mental health care,” “Staffing schools with a greater number of armed security personnel,” “Requiring individuals to obtain a license to purchase a firearm,” “Increasing the age limit for purchasing any firearm to 21,” “Increasing the number of metal detectors in schools,” “Allowing prayer in schools,” and “Requiring firearms to be stored locked and separated from ammunition.” Answer options for all policies included “(0) Not at all helpful,” “(1) Slightly helpful,” “(2) Moderately helpful,” “(3) Substantially helpful,” and “(4) Extremely helpful.” Participants could also indicate that they did not know or could refuse to answer the item.

### Analytic plan

In our primary analyses, we utilized a series of multivariate analyses of variances (MANOVAs) to examine between group differences in perceived levels of effectiveness for various policies with respect to preventing mass school shootings. For each of the two groups of policies, we conducted three MANOVAs comparing different groups—firearm ownership status (firearm owners vs non-firearm owners), sex (males vs females), and political affiliation (Democratic vs Independent vs Republican). In each case, partial eta squared served as the index of effect size.

## Results

Full sample characteristics are displayed in Table 1. Our sample was representative of the overall demographic characteristics of New Jersey adults. Participants were relatively equally likely to be male (47.6%) or female (51.6%) and the distribution was fairly equal across age groups. The majority of participants (60.7%) identified as White and 40.0% reported having earned a college or graduate degree. A minority of New Jersey residents (19.8%) reported firearm access in or around their homes and 41.1% identified as Democrats (vs 34.9% Independent and 24.0% Republican).

Overall, respondents believed requiring background checks on all firearm sales (m = 3.30, SD = 1.12), increasing funds for mental healthcare (m = 3.06, SD = 1.24), expanding the scope of background checks (m = 3.05, SD = 1.30), and requiring individuals to obtain a license to purchase a firearm (m = 2.86, SD = 1.43) would be the most helpful policies for preventing mass school shootings. In contrast, respondents viewed arming teachers and other school personnel (m = 1.25, SD = 1.52), allowing prayer in school (m = 1.40, SD = 1.68), decreasing the number of entrances at schools (m = 1.83, SD = 1.51), and requiring the firearms be stored locked and separated from ammunition (m = 2.12, SD = 1.64) as the policies that would be least helpful in preventing mass school shootings. These results are presented in Table 2.

**Table 2:** Average level of perceived efficacy for specific policies for preventing mass school shootings, ranked by level of perceived utility.

Policy	Level of perceived helpfulness of policy in preventing mass school shootings							
	Mean	SD	Not at all	Slightly	Moderately	Substantially	Extremely	Unsure
			% (n)	% (n)	% (n)	% (n)	% (n)	% (n)
Universal background checks	3.32	1.10	3.7 (18)	5.5 (27)	10.2 (49)	15.6 (75)	63.6 (307)	1.3 (6)
Increasing funding for mental healthcare	3.08	1.23	5.5 (27)	6.5 (33)	16.6 (83)	14.3 (71)	54.2 (270)	2.6 (13)
Increasing the scope of background checks	3.07	1.27	7.7 (37)	6.6 (32)	11.6 (56)	19.0 (92)	54.8 (265)	0.4 (2)
Requiring license to purchase firearms	2.85	1.44	12.3 (61)	6.4 (32)	13.8 (68)	14.2 (70)	49.2 (242)	3.8 (19)
Banning high-capacity magazines	2.72	1.57	17.2 (83)	8.4 (40)	7.9 (38)	14.4 (69)	48.7 (235)	2.8 (14)
Banning AR-15s & other “assault”-style weapons	2.71	1.62	20.0 (96)	5.3 (26)	9.9 (47)	9.6 (46)	52.5 (252)	2.6 (13)
Increase age requirment to 21 for firearm purchase	2.40	1.55	17.9 (88)	12.4 (61)	16.1 (80)	12.6 (62)	37.1 (184)	3.6 (18)
Adding more armed school security personnel	2.26	1.54	19.8 (97)	13.0 (64)	19.2 (94)	11.4 (56)	33.1 (162)	2.9 (14)
Adding more metal detectors in schools	2.24	1.47	15.7 (78)	19.2 (95)	18.7 (93)	14.2 (70)	29.8 (148)	2.1 (11)
Requiring secure firearm storage	2.09	1.65	27.2 (134)	11.8 (58)	13.0 (64)	11.1 (55)	31.8 (157)	4.8 (24)
Reducing the number of entrances as schools	1.82	1.52	27.1 (130)	16.8 (80)	20.6 (99)	9.4 (45)	22.2 (106)	3.9 (19)
Allowing prayer in schools	1.37	1.67	48.6 (239)	6.5 (32)	9.5 (47)	5.7 (28)	20.2 (99)	9.2 (45)
Arming teachers & other school personnel	1.24	1.52	48.0 (232)	15.0 (73)	11.1 (54)	6.2 (30)	15.8 (77)	3.8 (18)

Perceived utility scored as follows: Not at all = 0; Slightly = 1; Moderately = 2; Substantially = 3; Extremely = 4. Answers of “unsure” were not included in calculations of mean levels of perceived utility

### Firearm ownership

Firearm owners and non-firearm owners differed significantly on their views of the effectiveness of several policies with respect to their ability to help prevent mass school shootings (First set of policy questions: λ = .11, *p* < .001, _p_η^2^ = .89; second set of policy questions: λ = .13, *p* < .001, _p_η^2^ = .87; see Table 3). Firearm owners endorsed higher mean beliefs in the utility of providing teachers and other school personnel with firearms (1.61 vs 1.06, _p_η^2^ = .02) and increasing the number of armed security personnel at schools (2.49 vs 2.04, _p_η^2^ = .02). In contrast, non-firearm owners endorsed higher mean beliefs in the utility of requiring background checks on all firearm sales (3.51 vs 2.81, _p_η^2^ = .07), expanding the scope of background checks (3.29 vs 2.56, _p_η^2^ = .05), banning AR-15s and similar “assault”-style weapons (3.12 vs 1.85, _p_η^2^ = .11), banning high capacity magazines (3.13 vs 2.05, _p_η^2^ = .08), and requiring firearms be stored locked and separate from ammunition (2.37 vs 1.64, _p_η^2^ = .04).

**Table 3:** Multivariate analyses of variances (MANOVAs) examining between group differences on the perceived utility of various policies for preventing mass school shootings.

	Sex				Firearm ownership				Political affiliation				
	Male	Female	_p_η^2^	p	Yes	No	_p_η^2^	p	Dem	Ind	Rep	_p_η^2^	p
	N = 192	N = 212			N = 75	N = 301			N = 132	N = 131	N = 74		
Universal background checks	3.01	3.64	.08	<.001	2.81	3.51	.07	<.001	3.70_a_	3.24_b_	3.12_b_	.06	<.001
Expanded background checks	2.72	3.44	.08	<.001	2.56	3.29	.05	<.001	3.64_a_	2.86_b_	2.81_b_	.10	<.001
Fewer school entrances	1.74	1.87	.00	.398	1.88	1.81	.00	.736	1.64_a_	1.65_a_	2.32_b_	.04	.003
Arming teachers/school personnel	1.40	1.07	.01	.029	1.61	1.06	.02	.005	0.68_a_	1.05_a_	2.36_b_	.18	<.001
Banning AR-15s	2.04	3.33	.16	<.001	1.85	3.12	.11	<.001	3.55_a_	2.58_b_	1.91_c_	.16	<.001
Banning high-capacity magazines	2.20	3.32	.13	<.001	2.05	3.13	.08	<.001	3.44_a_	2.78_b_	2.03_c_	.12	<.001
Omnibus test	λ = .07,* p* < .001, _p_η^2^ = .93				λ = .11,* p* < .001, _p_η^2^ = .89				λ = .07,* p* < .001, _p_η^2^ = .93				

	Male	Female	_p_η^2^	p	Yes	No	_p_η^2^	p	Dem	Ind	Rep	_p_η^2^	p
	N = 281	N = 210			N =91	N = 291			N = 153	N = 121	N = 86		
Increase mental health funding	2.74	3.18	.03	.009	2.91	2.97	.00	.728	3.29_a_	2.81_b_	2.59_b_	.05	<.001
Increase armed school security	2.13	2.31	.00	.224	2.49	2.04	.02	.015	2.03_a_	2.22_ab_	2.72_b_	.03	.003
License to purchase firearm	2.74	3.01	.01	.049	2.77	2.99	.01	.188	3.29_a_	3.07_a_	2.19_b_	.10	<.001
Age 21 for all firearm purchases	2.30	2.57	.01	.070	2.41	2.49	.00	.623	2.94_a_	2.26_b_	2.24_b_	.05	<.001
More school metal detectors	1.91	2.53	.05	<.001	2.29	2.17	.00	.507	2.25	2.07	2.57	.02	.052
Allow prayer in school	1.27	1.41	.00	.389	1.28	1.27	.00	.972	1.05_a_	1.04_a_	2.14_b_	.08	<.001
Secure firearm storage	1.77	2.55	.06	<.001	1.64	2.37	.04	<.001	2.75_a_	1.79_b_	1.64_b_	.10	<.001
Omnibus test	λ = .10,* p* < .001, _p_η^2^ = .90				λ = .13, *p* < .001, _p_η^2^ = .87				λ = .10, *p* < .001, _p_η^2^ = .90				

In analyses examining differences by political affiliation, values within rows that do not share subscripts differ significantly from one another (*p* < .05).

### Sex

Males and females differed significantly on their views regarding the effectiveness of various policies (first set of policy questions: λ = .07, *p* < .001, _p_η^2^ = .93; second set of policy questions: λ = .10, *p* < .001, _p_η^2^ = .90; see Table 3). Males endorsed a greater belief in the value of providing teachers and other school personnel with firearms (1.40 vs 1.07, _p_η^2^ = .01). In contrast, females endorsed greater belief in the value of requiring background checks on all firearm sales (3.64 vs 3.01, _p_η^2^ = .08), expanding the scope of background checks (3.44 vs 2.72, _p_η^2^ = .08), banning AR-15s and other similar “assault”-style weapons (3.33 vs 2.04, _p_η^2^ = .16), banning high capacity magazines (3.32 vs 2.20, _p_η^2^ = .13), increasing funding for mental health (3.18 vs 2.74, _p_η^2^ = .03), requiring a license to purchase firearms (3.01 vs 2.74, _p_η^2^ = .01), increasing the number of metal detectors in schools (2.53 vs 1.91, _p_η^2^ = .05), and requiring firearms be stored locked and separate from ammunition (1.41 vs 1.27, _p_η^2^ = .002).

### Political affiliation

Respondents differed from one another on the basis of political affiliation in their belief in the utility of various policies in preventing mass school shootings (first set of policy questions: λ = .07, *p* < .001, _p_η^2^ = .93; second set of policy questions: λ = .10, *p* < .001, _p_η^2^ = .90; see Table 3). The three groups differed in their perceived value of requiring background checks be conducted for all firearm sales (_p_η^2^ = .06), expanding the scope of background checks (_p_η^2^ = .10), reducing the number of entrances at schools (_p_η^2^ = .04), providing teachers and other school personnel with firearms (_p_η^2^ = .18), banning AR-15s and similar “assault”-style weapons (_p_η^2^ = .16), banning high capacity magazines (_p_η^2^ = .12), increasing mental healthcare funding (_p_η^2^ = .05), increasing the number of armed security personnel at schools (_p_η^2^ = .03), requiring a license to purchase firearms (_p_η^2^ = .10), increasing the age limit for all firearm purchases to 21 (_p_η^2^ = .05), allowing prayer in schools (_p_η^2^ = .08), and requiring that firearms be stored locked and separate from ammunition (_p_η^2^ = .10).

For universal background checks, Democrats (3.70) endorsed higher perceived utility than did Independents (3.24, p = .001) and Republicans (3.12, *p* < .001). For expanding the scope of background checks, Democrats (3.64) endorsed greater perceived utility than did Independents (2.86, *p* < .001) and Republicans (2.81; *p* < .001). For reducing the number of entrances at schools, Republicans (2.32) endorsed greater perceived utility than did Democrats (1.64, p = .005) and Independents (1.65, p = .006). For providing teachers and other personnel with firearms, Republicans (2.36) reported higher perceived utility than Independents (1.05, *p* < .001) and Democrats (0.68, *p* < .001). For banning AR-15s and other “assault”-style weapons, Democrats (3.55) endorsed higher perceived utility than did Independents (2.58, *p* < .001), who in turn endorsed higher perceived utility than Republicans (1.91, p = .005). For banning high-capacity magazines, Democrats (3.44) endorsed higher perceived utility than did Independents (2.78, *p* < .001), who in turn endorsed higher perceived utility than Republicans (2.03, p = .001).

For increasing funding for mental healthcare, Democrats (3.29) endorsed higher perceived utility than did Independents (2.81, p = .004) and Republicans (2.59, *p* < .001). For increasing the number of armed security personnel at schools, Republicans (2.72) reported higher perceived utility than did Democrats (2.03, p = .002). For requiring a license to purchase a firearm, Democrats (3.29, *p* < .001) and Independents (3.07, *p* < .001) reported greater perceived utility than did Republicans (2.19). For increasing the age to 21 for the purchase of any type of firearm, Democrats (2.94) reported higher perceived utility than did Independents (2.26, *p* < .001) and Republicans (2.24, p = .001). For allowing prayer in school, Republicans (2.14) reported higher perceived utility than did Democrats (1.05, *p* < .001) and Independents (1.04, *p* < .001). Lastly, for requiring that firearms be stored locked and separate from ammunition, Democrats (2.75, p = .028) reported higher perceived utility than did Independents (1.79, *p* < .001) and Republicans (1.64, *p* < .001).

## Discussion

In this study, we sought to examine differences in perceived utility for policies to prevent school shootings among a sample of New Jersey residents. Our analyses produced three main findings. First, females were more likely to perceive policies like background checks, banning certain firearms and magazines, and requiring secure storage to be more effective while males perceived greater utility for arming school personnel. Second, firearm owners had lower perceived utility for policies related to background checks, banning certain firearms and magazines, and requiring secure storage. However, they perceived greater utility in policies that arm school personnel and increase armed school security. Finally, Democrats generally perceived the greatest level of utility for background checks, banning certain firearms and magazines, and enacting greater gun safety measures. Generally, Republicans demonstrated greater perceived utility for policies related to school hardening (reducing entrances, arming teachers and increasing security), and allowing prayer in school.

Our findings cohere with a large body of literature showing substantial variation in support for various firearm policies based on a wide range of demographic, political, and experiential factors (Crifasi et al. 2021a, 2022, 2021b; Dixon et al. 2020; Berryessa et al. 2022). Research generally indicates that, although there are clearly important differences in policy support across subgroups, there are far more similarities than differences in the support Americans demonstrate for many firearm policies (Barry et al. 2018; Crifasi et al. 2021a), including among firearm owners and non-firearm owners. Yet our results here are notable because they are derived from New Jersey, a state that is relatively liberal compared to most US states and has already enacted substantial firearm regulation (Giffords Law Center 2024). The findings underscore that, even in parts of the country with relatively stringent gun control and low rates of firearm injury and death, there remain important differences in perceived utility for specific policies, at least with respect to preventing school shootings. Such results highlight the need to ensure that conversations aimed at highlighting the evidence supporting (or not supporting) specific policies needs to be delivering in a manner—and through a range of media—capable of reaching and resonating with diverse communities rather than taking a one-size-fits-all approach our being housed within an echo chamber seen credible only by specific subgroups.

Although we focus here on subgroup differences, it is equally important to note where we found parity on particular policies. For instance, we found no statistically different perceptions of utility for policies related to reducing school entrances, requiring all firearm purchasers to be 21 years old, and allowing prayer in school across sex or firearm ownership groups. There were also no significant differences in perceptions of utility for increasing metal detectors in schools. Although certain policies may not be feasible due to a lack of evidence to support them, certain evidence-based regulations (e.g., requiring all purchasers to be 21+) can represent critical opportunities for strengthening firearm safety backed by a broader coalition of supporters (Morain and Crifasi 2019).

It is also worth noting that policies focused on reducing the number of entrances to schools, arming teachers and other school personnel, and allowing prayer in school were seen as particularly ineffective policies for preventing school shootings. Indeed, approaches centered on changing the physical environment of the school appeared to be seen as less effective by New Jersey residents overall. Notably, there was also limited perceived utility in mandating secure firearm storage despite the fact that a substantial percentage of school shooters acquire their firearms from a relative or peer. This discrepancy highlights that national conversations about secure firearm storage have failed to effectively persuade communities that limiting immediate access to firearms can impact the likelihood of firearm injury and death. Furthermore, the lack of perceived utility of school hardening is in direct contrast to the persistent discussion of such approaches by some elected officials and firearm rights advocacy groups, highlighting that dominant narratives disseminated within the media on this issue may not represent the views and beliefs of those theoretically represented by the individuals advocating for those approaches.

Our study has certain limitations that present opportunities for future research. First, the data are only representative of the adult population of New Jersey. Future researchers should aim to assess perceived utility of policies to reduce school shootings in diverse parts of the country and through nationally representative samples. Second, we were limited to examining certain policies and subgroups due to time and budgetary constraints for our surveying methods. For instance, we were unable to assess differences in perceived utility for policies related to extreme risk protection orders (ERPOs) or differences by regional location within New Jersey. Additionally, our sample size precluded analyses that considered the intersectionality of individual identities (e.g. political conservative vs politically liberal women). As such, our results reflect broad trends across groups and are unable to provide insight into the heterogeneity of any particular group. Future work should also consider neighborhood level factors rather than focusing exclusively on individual level variables. It should also be noted that the percengate of our sample living in homes with firearms was fairly low; however, this is representative of New Jersey communities. Finally, we were only able to assess the respondants’ own private perceived utility for certain policies and could not account for their perceptions of *others’* support for those same policies. Research suggests that supporters of gun safety policies often underestimate their peers’ support for the same regulations, shaping critical differences in public and private support that can affect regulatory adoptions (Dixon et al. 2020). Additional research on perceived utility of policies to reduce school shootings is needed that accounts for these critical dynamics.

Despite these limitations, our study provides critical insight into both the differences and similarities in perceived support for policies to reduce school shootings in New Jersey. A broad coalition of support across diverse sex, firearm ownership, and political groups will be necessary to meaningfully reduce gun violence both in New Jersey and throughout the rest of the country. As such, it is imperative that researchers and policymakers alike continue to identify possibilities for collaborative policy support to ensure progress towards the shared goal of greater safety in our schools.

## Data Availability

Data will be made available upon reasonable request of the corresponding author.

## Footnotes

1 We tested for between group differences in the two halves of the sample with respect to sex, age, racial identity, and political affiliation. The only significant difference noted was that a higher percentage of the half of the sample that answered the second set of policy questions was aged 18-34 (30.0% vs 22.2%; omnibus p = .011). Given that we did not compare the two halves of the samples to one another in any analyses, we did not include age as a covariate; however, the overall lack of meaningful differences between groups helps assure uniformity in analyses drawn from each half of the sample.

## References

[CR1] Barry CL, Webster DW, Stone E, Crifasi CK, Vernick JS, McGinty EE. Public support for gun violence prevention policies among gun owners and non–gun owners in 2017. Am J Public Health. 2018;108(63):878–81. 29771617 10.2105/AJPH.2018.304432 PMC5993389

[CR2] Benen S. The misguided, illegal, and ironic GOP push for school prayer. MSNBC. 2022. https://www.msnbc.com/rachel-maddow-show/maddowblog/misguided-illegal-ironic-gop-push-school-prayer-rcna32763. Accessed 10 Jul 2024

[CR3] Berryessa CM, Sierra-Arevalo M, Semenza DC. Portrayals of gun violence victimization and public support for firearm policies: an experimental analysis. J Exp Criminol. 2022;22:1–26. 10.1007/s11292-022-09517-x PMC11323076 39144402

[CR4] Burton AL, Pickett JT, Jonson CL, Cullen FT, Burton VS. Public support for policies to reduce school shootings: a moral-altruistic model. J Res Crime Delinq. 2021. 10.1177/0022427820953202. 10.1177/0022427820953202

[CR5] Giffords Law Center. New Jersey gun laws. https://giffords.org/lawcenter/gun-laws/states/new-jersey/.2023. Accessed 9 Jan 2024.

[CR6] Crifasi CK, Ward JA, McGinty EE, Webster DW, Barry CL. Public opinion on gun policy by race and gun ownership status. Prevent Med. 2021a;149: 106607. 10.1016/j.ypmed.2021.106607 PMC8273872 33984373

[CR7] Crifasi CK, Stone EM, McGinty EE, Barry CL. Differences in public support for gun policies between women and men. Am J Prevent Med. 2021b;60(1):e9-14. 10.1016/j.amepre.2020.06.028 PMC7859883 33341189

[CR8] Crifasi CK, Ward JA, McGinty EE, Barry CL, Webster DW. Public opinion on laws regulating public gun carrying. Prevent Med. 2022;159: 107067. 10.1016/j.ypmed.2022.107067 PMC11578283 35460721

[CR9] Dixon G, Garrett K, Susmann M, Bushman BJ. Public opinion perceptions, private support, and public actions of US adults regarding gun safety policy. JAMA Netw Open. 2020;3(12):e2029571. 33351084 10.1001/jamanetworkopen.2020.29571 PMC7756237

[CR10] Government of Canada. Firearms, accidental deaths, suicides and violent crime: An updated review of the literature with special reference to the Canadian situation (2022). https://www.justice.gc.ca/eng/rp-pr/csj-sjc/jsp-sjp/wd98_4-dt98_4/p2.html#:~:text=Canada's%20estimated%207.1%20million%20firearms,1998%2C%2052%2D53).

[CR11] Mancini C, Cook AK, Smith JC, McDougle R. Packing heat in the classroom: public support for “armed teacher” policy. J School Violence. 2020. 10.1080/15388220.2020.1786835. 10.1080/15388220.2020.1786835

[CR12] Marsh J. Eric adams: “When we took prayers out of schools, guns came into schools.” Politico. 2023. https://www.politico.com/newsletters/new-york-playbook-pm/2023/02/28/eric-adams-when-we-took-prayers-out-of-schools-guns-came-into-schools-00084832. Accessed 10 Jul 2024.

[CR13] Mascia J, Brownless C. How many guns are circulating in the U.S.? The Trace. 2024. https://www.thetrace.org/2023/03/guns-america-data-atf-total/. Accessed 2 Jul 2024

[CR14] Morain SR, Crifasi CK. Time to pull the trigger? Examining the ethical permissibility of minimum age restrictions for gun ownership and use. Prevent Med. 2019;118:205–9. 10.1016/j.ypmed.2018.11.003 30412741

[CR15] National Center for Education Statistics. Violent deaths at school and away from school, school shootings, and active shooter incidents. Condition of Education. U.S. Department of Education, Institute of Education Sciences. 2023 https://nces.ed.gov/programs/coe/indicator/a01 Accessed 9 Jan 2024.

[CR16] Pew Research Center. Amid a series of mass shootings in the U.S., gun policy remains deeply divisive. 2021. https://www.pewresearch.org/politics/2021/04/20/amid-a-series-of-mass-shootings-in-the-u-s--gun-policy-remains-deeply-divisive/. Accessed 10 Jul 2024.

[CR17] Schaeffer K. Key facts about Americans and guns. Pew Research. 2023. https://www.pewresearch.org/short-reads/2023/09/13/key-facts-about-americans-and-guns/Accessed 10 Jul 2024.

[CR18] Stanford L. Another state will let teachers carry guns. What we know about the strategy. Education Week. 2024. https://www.edweek.org/leadership/another-state-could-let-teachers-carry-guns-what-we-know-about-the-strategy/2024/04. Accessed 10 Jul 2024

[CR19] Tikkanen R, Fields K, Williams II, RD, Abrams MK. Mental health conditions and substance use: comparing U.S. needs and treatment capacity with those in other high-income countries. The Commonwealth Fund. 2020. https://digirepo.nlm.nih.gov/master/borndig/101769552/Tikkanen_mental_hlt_intl_comparison_db.pdf Accessed 3 Jul 2024.

[CR20] World Population Review. School shootings by country 2024. 2024 https://worldpopulationreview.com/country-rankings/school-shootings-by-country. Accessed 3 Jul 2024.

